# Dietary and Lifestyle Changes Before and After Diagnosis of Cardiovascular Disease

**DOI:** 10.1111/jhn.70317

**Published:** 2026-07-28

**Authors:** Aoi Suzuki, Yuri Ishii, Ribeka Takachi, Junko Ishihara, Kazumasa Yamagishi, Yoshihiro Kokubo, Isao Saito, Hiroshi Yatsuya, Isao Muraki, Hiroyasu Iso, Shoichiro Tsugane, Norie Sawada

**Affiliations:** ^1^ Department of Food Science and Nutrition Graduate School of Humanities and Sciences, Nara Women's University Nara Japan; ^2^ Division of Cohort Research National Cancer Center Institute for Cancer Control Tokyo Japan; ^3^ Graduate School of Environmental Health, Azabu University Kanagawa Japan; ^4^ Department of Public Health Medicine, Institute of Medicine, and Health Services Research and Development Center University of Tsukuba Ibaraki Japan; ^5^ Department of Public Health, Graduate School of Medicine Juntendo University Tokyo Japan; ^6^ Department of Preventive Cardiology National Cerebral and Cardiovascular Center Osaka Japan; ^7^ Department of Public Health and Epidemiology, Faculty of Medicine Oita University Oita Japan; ^8^ Department of Public Health and Health Systems, Nagoya University Graduate School of Medicine Showa‐ku, Nagoya‐shi Nagoya Japan; ^9^ Institute for Global Health Policy Research, Japan Institute for Health Security Shinjuku‐ku Tokyo Japan; ^10^ International University of Health and Welfare Graduate School of Public Health Tokyo Japan

**Keywords:** cardiovascular disease, diagnosis, dietary change, lifestyle change

## Abstract

**Background:**

Although the presence or absence of cardiovascular disease (CVD) recurrence is related to lifestyle habits, including diet, evidence for the maintenance of dietary improvement among participants with incident CVD after acute treatment is scarce.

**Aim:**

To determine changes in diet and other lifestyle factors in participants with incident CVD in the Japan Public Health Center‐based Prospective Study (JPHC study) over a 5‐year period before and after diagnosis by comparing changes with those in participants without a diagnosis of CVD.

**Methods:**

Participants were 68733 subjects aged 45–76 years at the baseline survey. Intakes of seven nutrients and 16 food groups were estimated using Food Frequency Questionnaires at the baseline survey and 5‐year follow‐up survey, and the amount of change was determined. Those diagnosed with CVD during the 5‐year follow‐up period were defined as participants with incident CVD (800 cases) and those not diagnosed were considered the non‐CVD controls. Differences in the change between the two groups over the 5‐year period were examined using the Mann‐Whitney U test and multivariate linear regression analysis. Changes in smoking status, body mass index (BMI), and physical activity were compared by logistic regression analysis.

**Results:**

CVD participants appeared to restrict sodium intake after diagnosis, and even after energy adjustment were more likely to avoid eating sodium, miso soup and pickles. In addition, they were less likely to eat beef and pork than controls. Significantly more CVD participants than controls stopped smoking and had decreased BMI.

**Conclusion:**

Participants with incident CVD showed modification in selective lifestyle factors, characterised by reduced sodium intake and smoking cessation.

## Summary

- We compared 5‐year dietary changes in participants with incident CVD with those in non‐patients.
- Participants had apparently restricted sodium intake, even after energy adjustment.
- Participants were more likely to stop smoking and to decrease their BMI.

## 1 Introduction

Although cardiovascular disease (CVD) remains a major cause of mortality worldwide, recent improvements in the accuracy of diagnosis and treatment, together with aging of the population, have increased survival rates, and the number of survivors with a history of CVD is increasing [1, 2]. Given that those with a history of CVD are at higher risk of cardiovascular events than those without [3, 4], preventing the recurrence of CVD is an important measure in extending healthy life expectancy.

Because the recurrence of CVD is related to lifestyle, including diet [5, 6, 7, 8, 9], personal behaviour change is considered an essential strategy in any recurrence prevention programme [10]. However, lifestyle improvement is strongly influenced by the patient's own decision‐making. In Japan, the Guidelines for Secondary Prevention of Myocardial Infarction (JCS2011) [6] provide dietary recommendations for such components as sodium, potassium, saturated fatty acids (SFA), polyunsaturated fatty acids, especially n‐3 polyunsaturated fatty acids, and alcohol. Although focused on primary prevention, the guideline also deals with stroke, for which it recommends a reduction in salt and alcohol; consumption of fruit and vegetables, and fish; and avoidance of SFA [11]. In the US, the guidelines of the American Heart Association/American Stroke Association [7] aim to prevent the recurrence of stroke by recommending the Mediterranean diet and reducing sodium intake. All guidelines [6, 7, 11] recommend smoking cessation, increasing physical activity, and keeping body weight within an appropriate range. Qualitative studies about motivations for lifestyle change report that improvement becomes possible when advice is received from professionals, and when the benefit of the change is understandable [12]. In reality, however, previous studies of dietary habits soon after onset among CVD patients in Western countries (US and Europe) show that compliance is poor [13, 14, 15, 16].

Indeed, little is known about whether CVD patients with potentially poor dietary habits can adopt and maintain a suitable diet even modestly after survival. Only two studies [17, 18] have compared dietary changes before and after hypertension or CHD, and only one of these [18] compared changes against a non‐diagnosed group as control.

Therefore, the aim of this study was to determine dietary changes in participants with incident CVD for 5 years before and after diagnosis in comparison with those who were not diagnosed.

## 2 Materials and Methods

### 2.1 Study Setting and Participants

The Japan Public Health Center‐based Prospective Study (JPHC study) was launched in 1990 (Cohort I) and 1994 (Cohort II) in 11 public health center (PHC) areas. 140,420 participants aged 40–69 years were included at the time of the initial self‐administered survey. After the initial survey, 5‐ and 10‐year follow‐up self‐administered surveys were conducted in 1995–1998 and 2000–2003, respectively, to update information on diet, lifestyle and health status. In the present study, we used data from these 5‐ and 10‐year follow‐up surveys because the questionnaires they used provided more comprehensive dietary intake information than the initial survey. Hence, the 5‐year survey will be referred to as “the baseline survey” and the 10‐year survey as “the follow‐up survey”. The JPHC study has been described in detail elsewhere [19].

**Figure 1:**
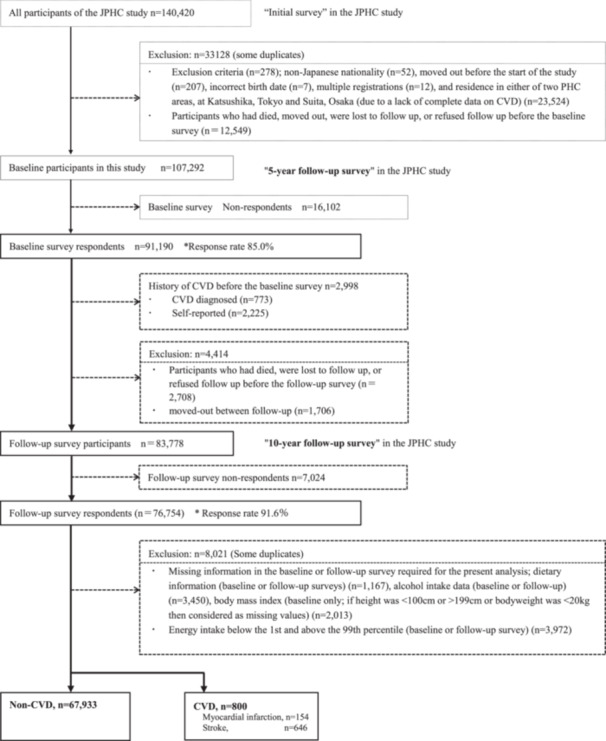
Flowchart of participants.

Patient flow is shown in Figure 1. Of the 140420 participants, we excluded those with non‐Japanese nationality (*n* = 52), moved out before the start of the study (*n* = 207), incorrect birth date (*n* = 7), multiple registrations (*n* = 12), lack of complete data on CVD incidence (two PHC areas: Katsushika, Tokyo and Suita, Osaka) (*n* = 23,524), or had died, moved out, or were lost to follow up or refused follow up before the baseline survey (*n* = 12,549) (some duplicates included). Of the remaining 107,292 participants, 91,190 participants responded to the baseline survey. We then excluded those with CVD diagnosed before the baseline survey date (*n* = 773); a self‐reported history of CVD at the baseline survey (*n* = 2225); had died, were lost to follow up or refused follow up before the follow‐up survey (*n*＝2708), or moved out during follow up (*n* = 1706). Of the remaining 83,778 participants, 76,754 participants responded to the follow‐up survey. We then further excluded participants with missing data on dietary intake (at either the baseline or follow‐up survey) (*n* = 1167), alcohol intake (at either the baseline or follow‐up survey) (*n* = 3450) or body mass index (BMI), with height < 100 cm or > 199 cm or bodyweight < 20 kg at baseline regarded as missing values (*n* = 2013); as well as those who reported extreme energy intake (below the 1st and above the 99th percentiles [20, 21] (< 825 kcal or > 5066 kcal for men and < 686 kcal or > 4472 kcal for women at baseline, or < 691 kcal or > 5243 kcal for men and < 549 kcal or > 4688 kcal for women at follow‐up survey) (*n* = 3972; some duplicates included). Finally, 68,733 participants (31,494 men and 37,239 women) were included in the study.

Participants were informed of the objectives of the study and told that completion of the survey questionnaire was regarded as providing consent to participate. The study was approved by the Institutional Review Boards of the National Cancer Center, Tokyo, Japan (approval number: 2001‐021). All procedures in this study were performed in accordance with the relevant ethical guideline regulations in Japan.

### 2.2 Food Frequency Questionnaire (FFQ)

Dietary intake was estimated from the validated FFQ used in the baseline and follow‐up surveys. Participants were asked about their usual intake of 138 food and beverage items over the past year. The FFQs contained nine frequency categories for food items ranging from “almost never” to “seven or more times per day.” Nine frequency choices for beverages ranged from “almost never” to “10 or more glasses per day”. Standard portion sizes were specified for each food item in the three amount choices of small (50% smaller than standard), medium (standard) and large (50% larger). The amount of foods and beverages consumed (g/day) was calculated from the responses. Energy and nutrient intakes were calculated using the Standard Tables of Food Composition in Japan 2015 (7th Revised Edition, December 2015) [22]. In this study, we compared changes in intake between the follow‐up survey and baseline survey for seven nutrients and 16 food groups. The validity and reproducibility of this procedure for estimating nutrients and food groups have been confirmed [23, 24, 25, 26, 27]. Spearman's rank correlation coefficients among subsamples of the cohorts for food groups between energy‐adjusted intake based on the FFQ and those based on 28‐day (or 14‐day for one public health centre area in Okinawa) dietary records for men and women were reported as valid: of the items covered in this study, correlation coefficients ranged from 0.42 for pork to 0.82 for ethanol in men, and from 0.30 for fruit to 0.76 for pickles in women. Correlation coefficients for these intakes of the two FFQs conducted at a 1‐year interval for men and women were also reported to confirm reproducibility: corresponding correlation coefficients ranged from 0.49 for pork to 0.79 for miso soup in men, and from 0.53 for SFA to 0.81 for miso soup in women.

### 2.3 Information on CVD Diagnosis and Survival

Information on survival status, including survival between the baseline and follow‐up surveys, was collected annually from the residential registries of each municipality in the study areas. Death certificates for persons in the residential registry are forwarded to the Japanese Ministry of Health Labor and Welfare and coded for inclusion in the national Vital Statistics. Residency registration and death registration are required by the Basic Residential Register Law and Family Registry Law, respectively, and are considered to be complete.

A total of 78 hospitals participated in the register of CVD events within the nine PHC areas. All hospitals were major hospitals capable of treating patients with acute coronary heart disease or stroke events. Physicians in the hospitals, PHCs or investigators reviewed the medical records of cohort participants at each hospital while blinded to patient lifestyle data and extracted clinical information including brain images, electrocardiograms, and cardiac enzymes into cohort‐specific registration forms. In this study, myocardial infarction and stroke were included as CVD because it has been established that dietary habits are a particular risk factor for these diseases [28], and because these diseases are characterised by acute attacks, patients are generally more aware of their symptoms than with other conditions, which is in turn thought to serve as a strong motivator for lifestyle change. Myocardial infarction was confirmed in the medical records according to the criteria of the Monitoring Trends and Determinants of Cardiovascular Disease (MONICA) project [28], which requires typical chest pain and evidence from electrocardiograms and/or cardiac enzymes. Cases with typical prolonged chest pain (> 20 min) but no electrocardiogram or cardiac enzyme confirmation were included in the myocardial infarction cases with a diagnosis of possible myocardial infarction. Stroke was confirmed according to the criteria of the National Survey of Stroke [29], which requires the presence of focal neurological deficits of sudden or rapid onset lasting at least 24 h or until death. Nearly all the registered hospitals provided CT and/or MRI imaging. Additional details of CVD diagnosis have been provided elsewhere [30]. Only the first myocardial infarction or stroke event during the follow‐up period was included. If a participant experienced both, only the first event was included.

### 2.4 Statistical Analysis

Dietary changes over the 5 years between the follow‐up and baseline surveys were calculated for each participant. Of 68733 participants included in the analysis, those diagnosed with CVD during the 5‐year follow‐up were defined as “participants with incident CVD”, and those not diagnosed with CVD during this follow‐up were defined as “non‐CVD controls”. Differences in the amount of dietary change over the 5 years between the two groups were analyzed by the Mann‐Whitney's U test. To determine the amount of dietary change in participants with incident CVD compared to non‐CVD controls, multivariate linear regression analyses were also performed with the dependent variable being the amount of dietary change and the independent variable being the presence or absence of a CVD diagnosis. The following information from the baseline survey was included as covariates: age (continuous), PHC area (nine areas), BMI (kg/m^2^) (< 18.5, 18.5–24.9, 25–29.9, or ≥ 30), living alone (yes/no), physical activity in metabolic equivalent task‐hours/day (METs‐h/day) (< 30, 30–34.9, 35–39.9, ≥ 40, or missing), smoking status (never, past, current < 20, or ≥ 20 cigarettes/day), and quintile of energy intake (except for analyses of energy intake). Energy intake was included as a covariate to determine whether the changes observed in other nutrients and food groups were attributable to changes in total dietary intake. Further, we used the Mann‐Whitney U test to analyze differences in intake between the two groups at baseline survey to evaluate the possibility of an effect on dietary changes by differences at the baseline survey. For items with differing intake between the two groups at the baseline survey, baseline intake was added as a covariate. In this context, the non‐standardised coefficient of the dietary variable in multivariate linear regression model (B) represents the amount of change in participants with incident CVD relative to that in non‐CVD controls. Because those taking warfarin are restricted from consuming vitamin K‐rich foods, a sensitivity analysis for dietary changes was conducted by excluding those taking warfarin at the follow‐up survey. Stratified analyses were also conducted regarding the use of medication for hypertension at the follow‐up survey and by a history of diabetes mellitus (DM) at the follow‐up survey. Additionally, we also conducted an analysis to compare non‐CVD control participants with participants with incident CVD matched at a 5:1 ratio using propensity scores and stratified by sex. Age, PHC area, BMI, living alone, physical activity in METs‐h/day, and smoking status at baseline were used as covariates.

Furthermore, multivariable logistic regression analysis was used to calculate the odds ratios (ORs) and 95% confidence intervals (CIs) of participants with incident CVD compared to non‐CVD controls for the lifestyle changes of smoking status, BMI, and physical activity, namely for smoking, those who were current smokers at baseline but had stopped at follow‐up; for BMI, those with a BMI ≥ 25.0 at baseline who had lost weight toward the ideal at follow‐up; and for physical activity, those who had increased or decreased by one activity category at follow‐up compared with that at baseline (divided into three categories of low, middle, and high), respectively. These analyses included age　(continuous), PHC area, and living alone (yes/no) as covariates.

Additionally, among participants with incident CVD, the magnitude of dietary change over 5 years in those who had improved their lifestyle was compared with that in those whose lifestyle has not changed (or decreased for physical activity). For smoking status, those who had stopped smoking at follow‐up were compared to those who had continued smoking; for BMI, among those who were ≥ 25.0 at baseline, those who had decreased toward the ideal at follow‐up were compared to those who had not changed; and for physical activity, the group that showed an increase in category (divided into three groups) at follow‐up from baseline were compared to the other groups of change. Mann‐Whitney's U test was used in the analysis of smoking status and BMI, and the Kruskal‐Wallis test in the analysis of physical activity. Due to the exploratory nature of this study, which evaluated a wide range of dietary and lifestyle factors, we did not apply formal corrections for multiple comparisons.

*P* values were 2‐sided, and statistical significance was determined as *p* < 0.05. All analyses were performed using SAS version 9.4 (SAS Institute Inc. Cary NC, USA).

## 3 Results

Of 68,733 participants included in the analysis, 800 (508 men and 292 women) were diagnosed with CVD during the 5‐year follow‐up period and classified as participants with incident CVD. Of these, 154 participants were diagnosed with myocardial infarction and 646 with stroke. Average time from diagnosis to the follow‐up survey was 2.2 years. Characteristics of participants at the baseline survey are shown in Table 1.

**Table 1:** Characteristics of participants at the baseline survey according to subsequent CVD diagnosis.

	Men					Women				
	Non‐CVD	CVD	*p* a	Myocardial infarction	Stroke	Non‐CVD	CVD	*p* a	Myocardial infarction	Stroke
No. of participants	30,986	508		107	401	36,947	292		47	245
Age (years, median)	56	61	**< 0.001**	58	61	56	61	**< 0.001**	62	61
[IQR]	[50.0–62.0]	[54.5–65.0]		[52.0–62.0]	[56.0–65.0]	[50.0–62.0]	[55.0–65.0]		[55.0–67.0]	[55.0‐65.0]
BMI (kg/m^2,^ %)										
< 18.5	2.6	2.4	0.663	1.9	2.5	3.4	1.4	**< 0.001**	4.3	0.8
≥ 18.5, < 25.0	67.8	67.5		60.8	69.3	66.4	60.3		59.6	60.4
≥ 25.0, < 30.0	27.3	27.2		34.6	25.2	26.8	32.2		31.9	32.2
≥ 30.0	2.2	3.0		2.8	3.0	3.4	6.2		4.3	6.5
Physical activity (METs‐h/day, %)										
< 30	33.5	33.9	0.498	45.8	30.7	32.0	30.8	0.477	29.8	31.0
≥ 30, < 35	18.1	21.1		16.8	22.2	24.2	21.9		19.2	22.5
≥ 35, < 40	22.0	19.5		14.0	21.0	27.8	31.9		38.3	30.6
≥ 40	23.5	22.1		18.7	22.9	13.3	11.6		8.5	12.2
Missing	2.9	3.5		4.7	3.2	2.8	3.8		4.3	3.7
Smoking status (%)										
Never	26.9	21.7	**< 0.001**	14.0	23.7	90.8	90.4	0.348	87.2	91.0
Past	25.7	19.0		15.0	20.2	1.3	0.7		0.0	0.8
Current	46.4	57.5		69.2	54.4	4.7	6.2		10.6	5.3
Missing	1.0	1.8		1.9	1.8	3.3	2.7		2.1	2.9
Living alone (%)	2.4	3.0	0.461	1.9	3.2	5.5	5.8	0.836	2.1	6.5
Public health centre area (%)										
Ninohe	11.0	10.6	0.549	8.4	11.2	12.2	14.7	0.277	4.3	16.7
Yokote	14.7	14.2		16.8	13.5	15.5	16.1		10.6	17.1
Saku	13.2	14.4		10.3	15.5	12.3	13.0		21.3	11.4
Okinawa	8.4	8.5		15.9	6.5	8.2	6.9		8.5	6.5
Mito	21.9	19.1		18.7	19.2	19.9	18.5		19.2	18.4
Nagaoka	3.4	3.4		1.9	3.7	3.3	3.8		4.3	3.7
Kochi	8.1	8.1		11.2	7.2	8.3	6.2		10.6	5.3
Nagasaki	8.9	11.0		11.2	11.0	9.9	11.3		12.8	11.0
Miyako	10.5	10.8		5.6	12.2	10.5	9.6		8.5	9.8
History of DM (%)	5.8	11.0	**< 0.001**	10.3	11.2	2.9	4.8	0.055	12.8	3.3
Medication (%)	32.5	50.4	**< 0.001**	41.4	52.9	34.7	62.0	**< 0.001**	68.1	60.8
Hypertension (%)	17.8	35.2	**< 0.001**	25.2	37.9	19.3	42.5	**< 0.001**	38.3	43.3
Dyslipidemia (%)	3.3	3.5	**< 0.001**	3.7	3.5	6.8	9.6	**< 0.001**	19.2	7.8

Abbreviations: BMI, body mass index; CVD, cardiovascular disease; DM, diabetes mellitus; IQR, interquartile range; METs‐h, metabolic equivalents task‐hours.

^a^ The Mann‐Whitney U test was used for the difference between participants with incident CVD, myocardial infarction or stroke and non‐CVD controls; *p* < 0.05 was considered significant and is presented in bold.

A greater decrease in the intake of energy, sodium and ethanol was seen in the participants with incident CVD than in the non‐CVD controls in both men and women in crude values (Table 2). Similarly, greater decreases were observed in participants with incident CVD for miso soup (men) and pickles (men and women), as well as in SFA (women) and beef and pork (men) (Table 2). On adjustment for covariates, including energy (Table 3), significantly greater decreases in participants were also seen for sodium (men, B = −218, *p* = 0.049) and ethanol (men, B = −15, *p* < 0.001). The significance of the results in crude values for miso soup (men), pickles (women), SFA (women), and beef and pork (men) remained unchanged after adjustment. With regard to fruit and vegetables, while a significantly smaller decrease in fruit intake in the CVD group compared with non‐CVD controls was seen only in men, a larger increase in participants with incident CVD compared with non‐CVD controls was not observed. These results did not change on exclusion of participants who took warfarin at the time of the follow‐up survey (n = 190) (data not shown). Further, results did not substantially change on stratification by use of medication for hypertension at the follow‐up survey (with medication, *n* = 17833) (data not shown), or by a reported history of DM at the follow‐up survey (DM, *n* = 4295) (data not shown) (characteristics at the follow‐up survey are shown in Supporting Information S1: Table S1). Finally, these results did not change on analysis with matched participants (data not shown).

**Table 2:** Changes in intakes of nutrients and food groups from baseline in participants with incident CVD (myocardial infarction or stroke) compared with non‐CVD controls.

	Non‐CVD			CVD				Myocardial infarction				Stroke			
	B‐line Med	Changes		B‐line Med	Changes			B‐line Med	Changes			B‐line Med	Changes		
		Median (IQR)			Median (IQR)		*p* a		Median (IQR)		*p* a		Median (IQR)		*p* a
**Men**	***n* = 30986**			***n* = 508**				***n* = 107**				***n* = 401**			
**Nutrients**															
Energy (kcal)	2126	−34	(−466, 384)	2111	−227	(−636, 290)	**< 0.001**	1929*	−184	(−504, 340)	0.233	2129	−252	(−676, 277)	**< 0.001**
SFA (g)	15.34	−1.45	(−6.74, 3.74)	14.84	−1.59	(−7.08, 3.94)	0.548	14.96	−1.81	(−9.30, 3.05)	0.381	14.82	−1.57	(−7.01, 4.42)	0.821
n‐3 PUFA (g)	2.31	−0.21	(−1.00, 0.58)	2.37	−0.30	(−1.08, 0.57)	0.255	2.10	−0.18	(−0.95, 0.39)	0.649	2.47	−0.36	(−1.10, 0.64)	0.295
Sodium (mg)	4668	−254	(−1592, 1033)	4723	−535	(−1852, 826)	**0.010**	4571	−537	(−1728, 615)	0.184	4860	−535	(−1961, 912)	**0.026**
Potassium (mg)	2607	−61	(−755, 642)	2579	−87	(−852, 667)	0.589	2453	66	(−687, 605)	0.572	2622	−138	(−857, 694)	0.369
Dietary fibre (g)	11.3	0.7	(−2.8, 4.4)	11.7	0.8	(−3.3, 4.8)	0.766	11.1	1.2	(−2.8, 5.1)	0.314	11.8	0.5	(−3.5, 4.8)	0.855
Ethanol (g)	23	0	(−9, 5)	23	−3	(−35, 0)	**< 0.001**	3*	0	(−7, 0)	**0.037**	30*	−10	(−36, 0)	**< 0.001**
**Food groups** (g)															
Rice	420	77	(−50, 140)	420	77	(−60, 124)	0.328	420	65	(−80, 120)	0.436	420	77	(−50, 126)	0.483
Miso soup	236	0	(−86, 64)	250	0	(−150, 38)	**< 0.001**	236	0	(−150, 29)	**0.042**	300	0	(−135, 38)	**0.003**
Fruits	143	−15	(−96, 56)	131	−6	(−92, 80)	0.051	141	−6	(−100, 107)	0.261	130	−6	(−86, 68)	0.105
Excl. Pickles, juice	93	−1	(−53, 56)	89	0	(−52, 83)	0.130	93	13	(−65, 105)	0.163	88	−1	(−49, 73)	0.322
Vegetables	174	−7	(−82, 67)	177	−6	(−88, 82)	0.459	153	7	(−61, 79)	0.173	181	−9	(−95, 82)	0.894
Excl. Pickles, juice	127	−1	(−56, 55)	134	4	(−63, 70)	0.161	121	12	(−46, 75)	0.063	137	3	(−65, 67)	0.533
Pickles	21	0	(−12, 13)	25	−2	(−20, 10)	**< 0.001**	24	−3	(−21, 8)	**0.023**	25	−2	(−20, 11)	**0.008**
FV juice	43	0	(−86, 0)	43	0	(−71, 0)	0.062	43	0	(−57, 0)	0.470	43	0	(−86, 0)	0.084
Seafood, salty	19	−3	(−15, 5)	20	−3	(−19, 6)	0.694	16	−3	(−14, 2)	0.771	20	−3	(−20, 7)	0.769
Seafood, not salty	57	−14	(−41, 10)	59	−17	(−47, 11)	0.193	52	−15	(−46, 5)	0.547	61	−17	(−47, 12)	0.247
Beef and pork	43	−12	(−36, 7)	43	−15	(−40, 4)	**0.029**	43	−16	(−44, 4)	0.117	43	−14	(−40, 4)	0.097
Soy products	52	−2	(−29, 26)	53	−4	(−35, 30)	0.372	41	0	(−30, 30)	0.639	54	−5	(−37, 30)	0.212
Dairy products	112	−13	(−99, 27)	100	−4	(−86, 70)	**0.006**	79	−3	(−77, 56)	0.201	104	−4	(−89, 78)	**0.015**
Green tea	300	0	(−274, 180)	300	0	(−300, 180)	0.670	326	0	(−300, 231)	0.381	300	0	(−300, 180)	0.352
Coffee (excl. canned)	26	0	(−26, 26)	26*	0	(−26, 0)	0.054	60*	0	(−94, 0)	**0.003**	26*	0	(−26, 0)	0.517
Sugary drink	143	−45	(−150, 14)	110*	−41	(−139, 14)	0.405	125	−47	(−168, 24)	0.849	107*	−29	(−131, 14)	0.299
**Women**	***n*** = **36947**			***n*** = **292**				***n*** = **47**				***n*** = **245**			
**Nutrients**															
Energy (kcal)	1801	−74	(−429, 282)	1772	−153	(−529, 194)	**0.005**	1920	−191	(−678, 170)	0.118	1767	−145	(−509, 201)	0.016
SFA (g)	16.00	−1.34	(−6.57, 3.70)	15.79	−2.21	(−8.23, 2.41)	**0.016**	17.16	−4.22	(−10.38, −0.05)	**0.015**	15.48	−2.03	(−7.56, 2.68)	0.118
n‐3 PUFA (g)	2.38	−0.19	(−0.93, 0.58)	2.24	−0.32	(−1.03, 0.50)	0.164	2.43	−0.36	(−0.92, 0.66)	0.853	2.22	−0.32	(−1.06, 0.49)	0.150
Sodium (mg)	4561	−264	(−1493, 930)	4433	−603	(−1889, 768)	**0.006**	4835	−838	(−2237, 310)	0.066	4233	−577	(−1834, 870)	**0.027**
Potassium (mg)	2787	−6	(−707, 723)	2654	−167	(−868, 798)	0.157	2958	−299	(−849, 767)	0.226	2587	−84	(−878, 833)	0.309
Dietary fibre (g)	13.2	1.0	(−2.8, 5.0)	12.9	−0.2	(−3.7, 5.2)	0.073	14.5	−0.9	(−3.3, 6.4)	0.615	12.7	−0.2	(−3.7, 5.0)	0.083
Ethanol (g)	0	0	(0, 0)	0	0	(0, 0)	**0.006**	0	0	(0, 0)	0.207	0	0	(0, 0)	**0.014**
**Food groups** (g)															
Rice	420	0	(−50, 65)	330	0	(−68, 60)	0.252	330	0	(−66, 60)	0.687	330	0	(−71, 60)	0.283
Miso soup	225	0	(−75, 44)	225	0	(−77, 75)	0.812	150*	0	(−75, 32)	0.841	225	0	(−78, 75)	0.863
Fruits	207	−18	(−114, 77)	210	−18	(−128, 97)	0.935	215	14	(−81, 100)	0.219	207	−21	(−138, 97)	0.532
Excl. Pickles, juice	154	0	(−74, 79)	163	4	(−90, 89)	0.480	174	19	(−65, 103)	0.274	153	−1	(−102, 85)	0.211
Vegetables	208	−3	(−82, 80)	196	−11	(−101, 85)	0.420	213	17	(−84, 79)	0.828	194	−12	(−104, 93)	0.329
Excl. Pickles, juice	162	0	(−60, 65)	148	7	(−67, 63)	0.764	161	12	(−63, 51)	0.989	146*	1	(−67, 65)	0.748
Pickles	24	0	(−14, 16)	27	−4	(−22, 13)	**< 0.001**	28	−3	(−18, 6)	0.122	26	−4	(−24, 13)	**0.002**
FV juice	43	0	(−86, 0)	43	0	(−86, 0)	0.922	43	0	(−57, 14)	0.136	43	0	(−86, 0)	0.448
Seafood, salty	21	−3	(−14, 6)	20	−5	(−19, 7)	0.075	28	−4	(−25, 9)	0.666	20	−5	(−19, 6)	0.079
Seafood, not salty	54	−12	(−36, 11)	50	−15	(−40, 12)	0.428	61	−11	(−37, 38)	0.409	49	−16	(−40, 6)	0.220
Beef and pork	38	−10	(−31, 6)	34	−10	(−36, 3)	0.352	46	−15	(−53, 0)	0.071	32*	−8	(−34, 4)	0.820
Soy products	55	0	(−28, 29)	52	−2	(−34, 31)	0.500	49	−2	(−41, 18)	0.475	53	−2	(−32, 32)	0.671
Dairy products	173	−18	(−118, 39)	160	−18	(−109, 53)	0.595	198	−60	(−196, 41)	0.172	157	−14	(−101, 56)	0.239
Green tea	394	0	(−274, 214)	420	−26	(−300, 206)	0.172	600	−26	(−300, 120)	0.202	394	0	(−300, 240)	0.350
Coffee (excl. canned)	26	0	(−26, 26)	26*	0	(−26, 0)	**< 0.001**	26	0	(−60, 0)	**< 0.001**	26*	0	(−26, 0)	**< 0.001**
Sugary drink	96	−33	(−107, 0)	100	−43	(−127, 14)	0.528	100	−43	(−150, 33)	0.937	100	−43	(−118, 14)	0.513

Abbreviations: B‐line med, median values of baseline survey; CVD, cardiovascular disease; excl, exclude; FV juice, fruits and vegetables juice; IQR, interquartile range; n, number of participants included in the analysis; PUFA, polyunsaturated fatty acid; SFA, saturated fatty acid.

^a^ The Mann‐Whitney *U* test was used for the difference in dietary change between participants with incident CVD, myocardial infarction, or stroke and non‐CVD controls; *p* < 0.05 was considered significant and is presented in bold.

* Significant difference (*p* < 0.05) in intake in the baseline survey between participants with incident CVD, myocardial infarction, or stroke and non‐CVD controls.

**Table 3:** Differences in the 5‐year change of intakes for participants with incident CVD compared with non‐CVD controls.

	CVD			Myocardial infarction			Stroke		
	B	95% CI	*p*	B	95% CI	*p*	B	95% CI	*p*
**Men**	***n* = 508**/31494			***n* = 107**/31093			***n* = 401/**31387		
**Nutrients**									
Energy (kcal)	−140	(−207, −73)	**< 0.001**	−108*	(−234, 17)	0.091	−164	(−239, −88)	**< 0.001**
SFA (g)	−0.58	(−1.58, 0.43)	0.262	−1.82	(−4.00, 0.35)	0.101	−0.24	(−1.37, 0.89)	0.677
n‐3 PUFA (g)	−0.03	(−0.17, 0.12)	0.721	−0.15	(−0.46, 0.17)	0.363	0.01	(−0.16, 0.17)	0.950
Sodium (mg)	−218	(−436, −1)	**0.049**	−169	(−638, 300)	0.480	−231	(−476, 13)	0.064
Potassium (mg)	25	(−90, 141)	0.665	79	(−170, 328)	0.535	11	(−119, 141)	0.866
Dietary fibre (g)	0.4	(−0.2, 1.1)	0.192	0.9	(−0.5, 2.3)	0.228	0.3	(−0.4, 1.0)	0.393
Ethanol (g)	−15	(−17, −12)	**< 0.001**	−8*	(−14, −3)	**0.002**	−15*	(−17, −12)	**< 0.001**
**Food groups** (g)									
Rice	−18	(−34, −1)	**0.035**	−18	(−54, 17)	0.312	−18	(−36, 1)	0.064
Miso soup	−22	(−35, −8)	**0.002**	−32	(−61, −4)	**0.028**	−19	(−34, −4)	**0.016**
Fruits	19	(2, 36)	**0.028**	11	(−26, 47)	0.567	21	(2, 40)	**0.028**
Excl. Pickles, juice	14	(0, 29)	0.051	14	(−17, 45)	0.376	15	(−2, 31)	0.080
Vegetables	12	(−4, 29)	0.127	37	(2, 71)	**0.037**	6	(−12, 24)	0.517
Excl. Pickles, juice	10	(−3, 23)	0.147	35	(7, 64)	**0.016**	3	(−12, 18)	0.697
Pickles	−3	(−7, 1)	0.150	−4	(−13, 5)	0.359	−3	(−7, 2)	0.248
FV juice	10	(0, 21)	**0.049**	2	(−20, 24)	0.855	12	(1, 24)	**0.033**
Seafood, salty	−2	(−5, 1)	0.190	−1	(−8, 6)	0.729	−2	(−6, 1)	0.194
Seafood, not salty	−1	(−7, 4)	0.663	−5	(−17, 8)	0.477	0	(−7, 6)	0.898
Beef and pork	−5	(−10, 0)	**0.038**	−11	(−22, −1)	**0.040**	−4	(−9, 2)	0.198
Soy products	−2	(−16, 12)	0.760	2	(−28, 32)	0.890	−3	(−19, 12)	0.674
Dairy products	9	(−14, 33)	0.431	12	(−38, 63)	0.634	9	(−18, 35)	0.520
Green tea	2	(−42, 45)	0.941	34	(−59, 128)	0.474	−7	(−56, 42)	0.779
Coffee (excl. canned)	−12*	(−23, −2)	**0.020**	−30*	(−53, −8)	**0.009**	−8*	(−19, 4)	0.204
Sugary drink	−11*	(−28, 7)	0.241	−5	(−53, 42)	0.827	−6*	(−26, 14)	0.547
**Women**	***n*** = **292**/37239			***n*** = **47**/36994			***n*** = **245**/37192		
**Nutrients**									
Energy (kcal)	−86	(−164, −8)	**0.031**	−110	(−304, 83)	0.265	−81	(−166, 4)	0.062
SFA (g)	−1.47	(−2.77, −0.18)	**0.026**	−1.79	(−5.00, 1.43)	0.276	−1.41	(−2.82, 0.00)	0.050
n‐3 PUFA (g)	−0.08	(−0.26, 0.10)	0.365	0.12	(−0.33, 0.56)	0.610	−0.12	(−0.32, 0.08)	0.226
Sodium (mg)	−306	(−898, 285)	0.310	−270	(−1742, 1203)	0.720	−313	(−959, 333)	0.342
Potassium (mg)	−102	(−260, 57)	0.209	21	(−373, 414)	0.918	−125	(−298, 48)	0.157
Dietary fibre (g)	−0.6	(−1.5, 0.3)	0.189	0.2	(−2.1, 2.5)	0.852	−0.8	(−1.8, 0.2)	0.130
Ethanol (g)	−1	(−2, 0)	0.139	−1	(−3, 1)	0.551	−1	(−2, 0)	0.176
**Food groups** (g)									
Rice	−14	(−29, 2)	0.087	−7	(−46, 31)	0.706	−15	(−32, 2)	0.088
Miso soup	1	(−14, 15)	0.944	−22*	(−54, 10)	0.177	1	(−15, 17)	0.912
Fruits	−2	(−29, 25)	0.899	68	(1, 135)	**0.048**	−15	(−44, 14)	0.317
Excl. Pickles, juice	−9	(−33, 15)	0.455	45	(−14, 105)	0.136	−20	(−46, 7)	0.143
Vegetables	−5	(−28, 18)	0.656	3	(−54, 59)	0.928	−7	(−31, 18)	0.600
Excl. Pickles, juice	1	(−19, 20)	0.936	−5	(−52, 43)	0.854	−1*	(−19, 17)	0.896
Pickles	−7	(−14, −1)	**0.016**	−6	(−21, 9)	0.454	−8	(−14, −1)	**0.021**
FV juice	9	(−5, 22)	0.199	35	(1, 69)	**0.041**	4	(−11, 19)	0.609
Seafood, salty	−1	(−5, 2)	0.452	2	(−8, 11)	0.707	−2	(−6, 2)	0.324
Seafood, not salty	−1	(−8, 6)	0.810	14	(−3, 30)	0.101	−4	(−11, 4)	0.328
Beef and pork	−8	(−14, −3)	**0.005**	−17	(−32, −3)	**0.020**	−4*	(−9, 1)	0.104
Soy products	−2	(−22, 18)	0.837	−2	(−52, 48)	0.931	−2	(−24, 20)	0.852
Dairy products	5	(−29, 38)	0.779	16	(−68, 99)	0.715	3	(−34, 39)	0.882
Green tea	−22	(−81, 37)	0.469	−94	(−241, 53)	0.212	−8	(−73, 56)	0.804
Coffee (excl. canned)	−30*	(−43, −18)	**< 0.001**	−42	(−77, −7)	**0.017**	−29*	(−43, −16)	**< 0.001**
Sugary drink	14	(−6, 34)	0.181	9	(−41, 60)	0.722	15	(−7, 37)	0.190

*Note:* n, number of participants included in this analysis who were diagnosed in 5 years/number of participants included in this analysis; B, multiple linear regression analysis was performed using the change of intake as dependent variable and presence of diagnosis (participants) as independent variable, including age (continuous), public health centre area, BMI (body mass index) in kg/m^2^ (< 18.5, 18.5–24.9, 25.0–29.9, ≥ 30), living alone (yes/no), physical activity in METs (metabolic equivalent task) ‐ hours/day (< 30, 30–34.9, 35.0–39.9, ≥ 40), smoking status (never, past, current < 20, current ≥ 20 cigarettes/day), and quintile of energy intake at baseline (excluded in the analysis of energy) as adjustment factors. Regression analysis for items that showed a significant difference (*p* < 0.05, *) in intake at the baseline survey between the groups for which median intakes at baseline were added as further covariates; changes are expressed as the difference between the baseline intake and follow‐up survey intake according to the formula (follow‐up survey intake − baseline survey intake); *p* < 0.05 was considered significant and is shown in bold.

Abbreviations: B, difference in intake change compared with non‐CVD controls; CI, confidence interval; CVD, cardiovascular disease; excl, exclude; FV juice, fruits and vegetables juice; PUFA, polyunsaturated fatty acid; SFA, saturated fatty acid.

Table 4 shows changes in lifestyle between the baseline and follow‐up surveys in participants with incident CVD compared with non‐CVD controls. Among current smokers at the baseline survey, the number who stopped smoking was greater in the participants with incident CVD in both men (OR, 8.3; 95% CI, 6.4–10.7) and women (OR, 7.2; 95% CI, 2.5–20.7). The number who decreased a BMI category towards an ideal BMI was also greater in the participants with incident CVD in both men (OR, 1.5; 95% CI, 1.0–2.1) and women (OR, 1.6; 95% CI, 1.0–2.4). Inconsistent with these findings, however, the number of patients who decreased a category of physical activity was greater (men: OR, 2.2; 95% CI, 1.7–2.8; women: OR, 2.8; 95% CI, 1.9–4.0) and the number who increased a category was significantly smaller for the participants with incident CVD (men: OR, 0.5; 95% CI, 0.4–0.7; women: OR, 0.5; 95% CI, 0.4–0.8). Among participants, those who stopped smoking reduced their sodium and beef and pork intake more than those who continued smoking in both men and women (Supporting Information S1: Table S2). Those whose BMI decreased toward the ideal also reduced their sodium and beef and pork intake more than those whose BMI category did not change for men. In contrast, however, there was no association between improved physical activity and improved diet.

**Table 4:** Lifestyle changes between the baseline and follow‐up surveys in participants with incident CVD relative to the non‐CVD controls.

	non‐CVD		CVD				Myocardial infarction				Stroke			
	*n* a	%^b^	*n*	%^b^	OR	95% CI	*n* a	%^b^	OR	95% CI	*n* a	%^b^	OR	95% CI
**Men**														
Stopped smoking^c^	2434/14,255	17.1	191/290	65.9	**8.3**	**(6.4, 10.7)**	62/73	84.9	**29.0**	**(15.1, 55.5)**	129/217	59.5	**5.9**	**(4.5, 7.8)**
Decreased BMI category^d^	2130/8676	24.6	50/148	33.8	**1.5**	**(1.0, 2.1)**	14/39	35.9	1.7	(0.9, 3.3)	36/109	33.0	1.4	(0.9, 2.1)
Decreased physical activity category	7733/16,882	45.8	201/297	67.7	**2.2**	**(1.7, 2.8)**	32/49	65.3	**2.2**	**(1,2, 3.9)**	169/248	68.2	**2.2**	**(1.6, 2.8)**
Increased physical activity category	8323/17,144	48.6	76/244	31.2	**0.5**	**(0.4, 0.7)**	19/61	31.2	**0.5**	**(0.3, 0.8)**	57/183	31.2	**0.5**	**(0.4, 0.7)**
**Women**														
Stopped smoking^c^	458/1705	26.9	13/18	72.2	**7.2**	**(2.5, 20.7)**	4/5	80	10.8	(1.1, 101.8)	9/13	69.2	**6.3**	**(1.9, 21.0)**
Decreased BMI category^d^	2398/10,400	23.1	35/104	33.7	**1.6**	**(1.0, 2.4)**	5/15	33.3	1.5	(0.5, 4.3)	30/89	33.7	**1.6**	**(1.0, 2.5)**
Decreased physical activity category	10,498/2,0342	51.6	139/178	78.1	**2.8**	**(1.9, 4.0)**	23/28	82.1	**3.7**	**(1.4, 9.8)**	116/150	77.3	**2.6**	**(1.8, 3.9)**
Increased physical activity category	9630/19,188	50.2	41/124	33.1	**0.5**	**(0.4, 0.8)**	4/20	20.0	**0.3**	**(0.1, 0.9)**	37/104	35.6	**0.6**	**(0.4, 0.9)**

*Note:* Significant values were shown in bold.

Abbreviations: BMI, body mass index; CI, confidence intervals; CVD, cardiovascular disease; OR, odds ratio compared with non‐CVD controls as reference, and adjusted for age (continuous), public health centre area, and living alone (yes/no).

^a^ Number of participants whose category was moved to the specified direction/number of participants whose category was not moved and was moved in the specified direction.

^b^ (Number of participants whose category was moved to the specified direction/number of participants whose category was not moved and was moved in the specified direction) ×100.

^c^ From current smokers at baseline to past smokers at follow‐up survey.

^d^ From ≥ 30.0 at baseline to 18.5–25.0 or 25.0–30.0 at follow‐up survey or from 25.0 to 30.0 at baseline to 18.5–25.0 at follow‐up survey.

## 4 Discussion

In this prospective study among middle‐aged Japanese, we found that participants with incident CVD decreased their intake of energy, ethanol, and sodium and sodium‐related foods compared to non‐CVD controls. In addition, participants with incident CVD also showed decreased intake of SFA in women and beef and pork in men. These changes were in the direction of adherence to guideline recommendations [6, 7, 11]. Although a smaller reduction in fruit intake in the CVD group compared with non‐CVD controls was observed only in men, a larger increase in fruit and vegetable intake was not observed. Compared to non‐CVD controls, participants with incident CVD showed high ORs of smoking cessation and a decrease in BMI category toward the ideal in both men and women. With regard to physical activity, however, a trend toward compliance with the guidelines was not observed.

Inconsistent with our results, previous reports on dietary habits after the occurrence of CVD showed that compliance was poor [13, 14, 15, 16]. While these studies [13, 14, 15, 16] evaluated the diet for up to 3 years after the onset of CVD, our study captured dietary changes for up to 5 years (range: 1 day to 5 years) after CVD diagnosis. Aburto et al. [17] examined changes in sodium, potassium, and sodium‐to‐potassium ratio intake before and within two to 4 years after diagnosis. Similar to our results, they showed an average decrease in sodium for men, despite including hypertension patients only with no control group. Similarly to our study, only one other study – conducted in the Netherlands [18] – compared 5‐year changes in diet and other lifestyle factors before and after a CHD diagnosis using a non‐diagnosed group as control. Unlike our study, however, this study reported no significant difference in changes in SFA, fruit, vegetables, or fish intake. CHD in that study was self‐reported, however, and participants were younger (aged 20–59 years) than in our study and sample size was smaller (*n* = 6386, 403 cases). Information on a diagnosis of CVD in our study was more accurate because it was based on registration [30]. Further, our study had a large number of participants, which may have been sufficient to detect differences in change.

Among reports of CVD patients after onset [13, 14, 15, 16], one study [13] reported the lifestyle habits of patients: of those who had smoked before developing CVD, a third of men and half of women continued to smoke after developing CVD, and of those, 29% of men and 38% of women reported inadequate physical activity. These results are similar to our present results, except for smoking among women. Because very few of the participants with incident CVD and current smokers in our baseline survey were women (6.2% in our study vs. 23% in the previous study [13]), the results may not have been consistent. Manschot et al. [18] also reported that participants with incident CHD were more likely to stop smoking compared to those without CHD, but saw no differences in change in BMI (also in energy intake) or physical activity between the two groups. This result for smoking status is consistent with our present results, but the results for BMI and physical activity are not. This discrepancy regarding BMI may be because a decrease in BMI to the ideal in our study was influenced by a larger decline in energy intake. The decrease in BMI in our study may be attributable the overall decrease in intake. Also, it remains possible that the decrease in BMI may have been due to disease‐related loss of muscle mass. Further, possible reasons for the discrepancy in results for CVD regarding physical activity, which tended to decrease in our study, may be that the participants in the previous study [18] were younger than those in our study. It is possible that many patients in that study were able to recover to the point where they were able to maintain their physical activity levels. A second reason may be that our study included a substantial number of stroke patients, who are more likely to have sequelae such as gait disturbance [31]. Also, since gait disturbance can also occur in CHD, it is reasonable that the proportion of myocardial infarction patients with a decrease in physical activity in this study was greater than in the non‐CVD control. Furthermore, our analysis of dietary changes in combination with lifestyle changes suggested that those with improved lifestyle habits other than diet were also more likely to have improved some aspects of their diet. In studies on factors which motivate lifestyle change, patients have reported that improvement becomes more likely when they receive advice from professionals, and understand the benefits of the changes [12]. The opportunity to receive advice from a nutrition expert through universal health insurance may motivate these changes. Additionally, Näslund et al. [32] reported that in participants who had CVD risk factors, those who were provided with pictorial information on atherosclerosis obtained from carotid ultrasounds showed a greater reduction in CVD risk factor score at 1‐year follow‐up than those who were not provided with this information. Awareness of the disease itself can be expected to induce further improvements in lifestyle, including diet. Also, issues that require a focus on long‐term dietary management (items that did not change or for which change was not sustained, despite guideline recommendations) were identified. For example, contrary to guideline recommendations, no increase in fruit and vegetable intake was observed. Accordingly, attention should perhaps be focused on aspects such as increasing fruit and vegetable intake.

While several statistically significant changes were observed in dietary intake, it should be noted that the absolute magnitudes of these changes were relatively modest. However, from a public health perspective, even small shifts in the distribution of lifestyle factors across a population of participants may contribute to a cumulative reduction in the risk of recurrence. Accordingly, future evaluation of the cumulative effects of the minute changes in intake identified in this study may be warranted.

This study has some limitations. First, all participants had responded to the follow‐up survey, and those who were not well enough to respond or who had died – in other words those with more severe disease – were excluded. Our results might therefore underestimate the degree of lifestyle change in participants. In fact, on comparison of characteristics and intakes at the baseline survey of this study between participants with incident CVD and excluded persons who did not respond to the follow‐up survey but were diagnosed with CVD in the 5‐year follow up showed a greater proportion of participants with a history of DM among the diagnosed but excluded persons, with corresponding values of 5.8% and 15.2% for men and 2.9% and 12.3% for women, respectively. Furthermore, this exclusion suggests a collider bias, indicating that both the severity of CVD and health awareness likely influenced continued participation in the study. Consequently, our findings may primarily reflect changes in lifestyle among participants with incident CVD who had relatively mild symptoms and high health literacy. Caution is therefore warranted when generalising these results to more severe cases or those with multiple comorbidities.

Second, the validity and reproducibility of the FFQ used in this study was examined in healthy people. Given that case groups report their diet more accurately than control groups because of recall bias [33], we cannot deny the possibility that the amount of dietary change in 5 years was overestimated in participants with incident CVD compared to non‐CVD controls. However, a similar report on changes in the diet of cancer survivors from the JPHC study [20] conducted in the same way as this study found almost no dietary change before and after diagnosis compared to non‐cancer controls, including for pickled vegetables. Therefore, positivity for disease does not necessarily mean that the amount of change was overestimated. One reason for the lack of change in most items among cancer survivors is that nutritional advice for cancer patients is primarily designed to treat the loss of appetite associated with the side effects of treatment [34].

Third, the potential for “social desirability bias” caused by potential differences in self‐reports following diagnosis should be considered. However, it is noteworthy that we observed no significant increases in the intake of vegetables, fruits, or fish—items typically emphasised in dietary counselling. If the results were solely driven by a reporting bias to provide socially desirable answers, a more universal improvement across all healthy food groups would be expected. Instead, the selective nature of the reported changes in this study suggests that our findings likely reflect actual behavioural shifts rather than a uniform reporting bias.

Fourth, the interval between CVD diagnosis and follow‐up assessment was heterogeneous. In this study, behavioural changes were assessed at a single time point after diagnosis, ranging from immediately after the event to several years later. It is well‐recognised that behavioural responses may differ between short‐term adoption and long‐term maintenance [35]. To account for this heterogeneity in the timing of the post‐diagnosis assessment, we further examined the interaction between CVD onset and time elapsed since diagnosis as a continuous variable. The results showed no interaction terms with *p* < 0.05. In addition, we conducted a stratified analysis based on the duration between diagnosis and follow‐up (one to 2 years and three to 4 years), and again found no differences based on duration. We therefore consider that any differences in the degree of change over time since diagnosis can be considered negligible within a 5‐year period.

Finally, we were unable to examine in detail what specific kinds of physical activity had changed, such as sitting or walking. This is because the questionnaire items used to calculate physical activity levels between the baseline and follow‐up surveys differed, and we were unable to verify the validity of the specific items. We therefore evaluated changes in each category separately (classified into low, moderate, and high) and determined that comparisons other than total METs were inappropriate. Clarification of the details of changes in physical activity is important for understanding behavioural changes in patients with CVD and should be addressed in future studies.

## 5 Conclusion

In conclusion, a diagnosis of CVD appeared to influence specific lifestyle changes among patients whose disease condition was likely relatively mild. These changes included reducing salt intake and quitting smoking. In contrast, however, no increase was seen in vegetable and fruit intake, and physical activity levels decreased.

## Author Contributions

Conceptualization and design: All authors. Data curation: Kazumasa Yamagishi, Yoshihiro Kokubo, Isao Saito, Hiroshi Yatsuya, Isao Muraki, Hiroyasu Iso, Shoichiro Tsugane, and Norie Sawada. Formal analysis: Aoi Suzuki and Yuri Ishii. Funding acquisition: Shoichiro Tsugane and Norie Sawada. Writing – original draft: Aoi Suzuki and Ribeka Takachi. Writing – review and editing: All authors. Supervision: Ribeka Takachi, Kazumasa Yamagishi, and Norie Sawada. Project administration: Norie Sawada. Approval of the final version text: All authors.

## Ethics Statement

The study was approved by the Institutional Review Boards of the National Cancer Center, Tokyo, Japan (approval number: 2001‐021). All procedures in this study were performed in accordance with the relevant ethical guideline regulations in Japan.

## Conflicts of Interest

Shoichiro Tsugane reports joint research funds outside of this work from Ajinomoto and Meiji Corporations. The other authors declare no conflicts of interest.

## Supporting information


Supporting File

## Data Availability

For information on how to apply to gain access to JPHC data, please follow the instructions at https://epi.ncc.go.jp/en/jphc/805/.
