# Supplementary material for: Dietary and Lifestyle Changes Before and After Diagnosis of Cardiovascular Disease
Source: J Hum Nutr Diet. 2026 Jul 28;39(4):e70317. doi: 10.1111/jhn.70317 (PMC13411027; doi:10.1111/jhn.70317)
Supplement: Supplementary file 1 — Supporting File [file JHN-39-0-s001.docx]

Supplemental Table 1. Characteristics of participants at follow-up survey according to previous CVD diagnosis

|  | Men | | | |  | Women | | | |
| --- | --- | --- | --- | --- | --- | --- | --- | --- | --- |
|  | **Non-CVD** | **CVD** |  |  |  | **Non-CVD** | **CVD** |  |  |
|  |  |  | Myocardial  infarction | Stroke |  |  |  | Myocardial  infarction | Stroke |
| No. of participants | 30,986 | 508 | 107 | 401 |  | 36,947 | 292 | 47 | 245 |
| BMI (kg/m^2,^ %) |  |  |  |  |  |  |  |  |  |
| <18.5 | 4.7 | 8.3 | 1.9 | 10.0 |  | 5.7 | 5.5 | 4.3 | 5.7 |
| ≥18.5, <25.0 | 64.8 | 62.2 | 62.6 | 62.1 |  | 63.3 | 60.3 | 66.0 | 59.2 |
| ≥25.0, <30.0 | 28.1 | 28.0 | 33.6 | 26.4 |  | 27.5 | 27.7 | 23.4 | 28.6 |
| ≥30.0 | 2.4 | 1.6 | 1.9 | 1.5 |  | 3.6 | 6.5 | 6.4 | 6.5 |
| Physical activity (METs-h/day, %) | | | | |  |  |  |  |  |
| <30 | 61.9 | 78.9 | 78.5 | 79.1 |  | 68.5 | 86.6 | 89.4 | 86.1 |
| ≥30, <35 | 20.2 | 13.0 | 16.8 | 12.0 |  | 19.8 | 8.6 | 8.5 | 8.6 |
| ≥35, <40 | 12.3 | 6.1 | 4.7 | 6.5 |  | 7.9 | 2.1 | 0.0 | 2.5 |
| ≥40 | 5.6 | 2.0 | 0.0 | 2.5 |  | 3.8 | 2.7 | 2.1 | 2.9 |
| Missing | 0.0 | 0.0 | 0.0 | 0.0 |  | 0.0 | 0.0 | 0.0 | 0.0 |
| Smoking Status (%) | |  |  |  |  |  |  |  |  |
| Never | 26.2 | 22.2 | 15.9 | 23.9 |  | 92.3 | 90.8 | 87.2 | 91.4 |
| Past | 32.3 | 55.9 | 72.9 | 51.4 |  | 2.4 | 5.1 | 8.5 | 4.5 |
| Current | 40.2 | 20.5 | 10.3 | 23.2 |  | 4.1 | 2.7 | 4.3 | 2.5 |
| Missing | 1.3 | 1.4 | 0.9 | 1.5 |  | 1.2 | 1.4 | 0.0 | 1.6 |
| Living alone (%) | 3.4 | 2.6 | 1.9 | 2.7 |  | 7.7 | 6.2 | 8.5 | 5.7 |
| History of DM (%) | 8.2 | 18.1 | 19.6 | 17.7 |  | 4.4 | 10.3 | 17.0 | 9.0 |
| Medication (%) | 40.4 | 89.6 | 95.3 | 88.0 |  | 43.1 | 85.3 | 87.2 | 84.9 |
| Hypertension (%) | 24.6 | 63.4 | 45.8 | 68.1 |  | 26.2 | 67.8 | 55.3 | 70.2 |
| Dyslipidemia (%) | 5.2 | 16.3 | 29.0 | 13.0 |  | 11.6 | 20.2 | 34.0 | 17.6 |

Abbreviations, CVD, cardiovascular disease; BMI, body mass index; METs-h, metabolic equivalents task-hours; DM, diabetes mellitus.

Supplemental Table 2. Changes in some nutrients and food groups from the baseline survey for participants with incident CVD stratified by status of change of smoking status, BMI, and physical activity

|  | By change in physical activity^a^ | | |  |  | By change in smoking status^b^ | |  |  | By change in BMI  towards ideal^c^ | |  |
| --- | --- | --- | --- | --- | --- | --- | --- | --- | --- | --- | --- | --- |
|  | Decreased | Remained | Increased |  |  | Continued | Stopped |  |  | Remained | Decreased |  |
|  | median | median | median | P^d^ |  | median | median | P^e^ |  | median | median | P^e^ |
| **Men, n** | 201 | 213 | 76 |  |  | 99 | 191 |  |  | 98 | 50 |  |
| Nutrients |  |  |  |  |  |  |  |  |  |  |  |  |
| Energy (kcal) | -346 | -198 | -46 | **0.049** |  | -159 | -266 | 0.741 |  | -108 | -460 | **0.010** |
| SFA (g) | -2.73 | -0.90 | -1.11 | 0.393 |  | -0.35 | -2.44 | 0.122 |  | -0.98 | -3.82 | 0.111 |
| Sodium (mg) | -697 | -628 | -20 | 0.109 |  | -453 | -629 | 0.538 |  | -309 | -1,145 | **0.027** |
| Ethanol (g) | -5 | -3 | 0 | 0.217 |  | -5 | -3 | 0.999 |  | -4 | 0 | 0.221 |
| Food groups (g) |  |  |  |  |  |  |  |  |  |  |  |  |
| Miso soup | 0 | 0 | -32 | 0.732 |  | 0 | 0.0 | 0.236 |  | -1 | -38 | 0.184 |
| Fruits | -15 | -5 | -14 | 0.777 |  | -23 | -3.5 | 0.211 |  | 27 | -61 | **<.001** |
| Vegetables | -14 | -8 | 11 | 0.772 |  | -10 | -17.6 | 0.970 |  | 12 | -26 | 0.135 |
| Pickles | -2 | -3 | -1 | 0.726 |  | -3 | -4.0 | 0.330 |  | -1 | -4 | **0.039** |
| FV juice | 0 | 0 | -43 | 0.117 |  | 0 | 0.0 | 0.131 |  | 0 | 0 | 0.302 |
| Beef and pork | -18 | -12 | -14 | 0.582 |  | -12 | -16 | 0.149 |  | -22 | -26 | 0.697 |
| Coffee (excl. canned) | 0 | 0 | 0 | 0.257 |  | 0 | 0.0 | 0.916 |  | 0 | 0 | 0.433 |
|  |  |  |  |  |  |  |  |  |  |  |  |  |
| **Women, n** | 139 | 101 | 41 |  |  | 5 | 13 |  |  | 69 | 35 |  |
| Nutrients |  |  |  |  |  |  |  |  |  |  |  |  |
| Energy (kcal) | -223 | -98 | -137 | **0.030** |  | 106 | -156 | 0.085 |  | 146 | 129 | 0.464 |
| SFA (g) | -3.38 | -1.95 | -0.05 | **0.039** |  | 4.33 | -4.30 | 0.402 |  | -3.38 | -1.43 | 0.160 |
| Sodium (mg) | -892 | -349 | -273 | 0.078 |  | 2395 | -571 | 0.460 |  | -640 | -232 | 0.225 |
| Ethanol (g) | 0 | 0 | 0 | 0.116 |  | 0 | 0 | 0.417 |  | 0 | 0 | 0.558 |
| Food groups (g) |  |  |  |  |  |  |  |  |  |  |  |  |
| Miso soup | 0 | 0 | 0 | 0.805 |  | -44 | 5 | 0.459 |  | 0 | 0 | 0.332 |
| Fruits | -39 | -9 | 10 | 0.235 |  | 90 | 23 | 0.257 |  | -1 | -27 | 0.447 |
| Vegetables | -17 | -11 | 32 | 0.265 |  | 33 | 57 | 0.961 |  | 12 | 19 | 0.618 |
| Pickles | -8 | -2 | 1 | 0.175 |  | -2 | -2 | 0.402 |  | -5 | -2 | 0.511 |
| FV juice | 0 | 0 | -43 | 0.783 |  | 0 | 0 | 0.880 |  | 0 | -43 | 0.483 |
| Beef and pork | -8 | -10 | -12 | 0.679 |  | 2 | -15 | 0.460 |  | -21 | -8 | 0.222 |
| Coffee (excl. canned) | 0 | 0 | 0 | 0.654 |  | -300 | 0 | 0.127 |  | 0 | 0 | 0.199 |

Abbreviations, median, median of change; FV juice, fruits and vegetables juice; excl, exclude; n, number of participants included in the analysis; ^a^ Included in all participants who diagnosed with CVD during five years; ^b^ included in smokers at the baseline survey who diagnosed with CVD during five years; ^c^ included in participants who overweight or obese at the baseline survey and diagnosed with CVD during five years; ^d^ they were calculated using Kruskal-Wallis test; ^e^ calculated using the Mann-Whitney's U test; P<0.05 was considered significant and is presented in bold.
